# Microbial Diversity Across Chemolithotrophic and Phototrophic Biofilms in Cold Sulfur Springs

**DOI:** 10.1002/mbo3.70223

**Published:** 2026-02-10

**Authors:** David M. Frings, James M. Mellinger, Kevin M. Drace

**Affiliations:** ^1^ Department of Biological and Environmental Sciences Samford University Birmingham Alabama USA

**Keywords:** cold sulfur springs, microbial ecology, sulfur metabolism, sulfur‐oxidizing bacteria

## Abstract

Sulfur‐rich environments host specialized microbial communities that drive key biogeochemical processes, particularly sulfur cycling. While sulfur‐oxidizing microbiota from hydrothermal vents and volcanic systems are well studied, microbial communities in cold terrestrial sulfur springs remain less understood. In this study, we used 16S rRNA gene sequencing to examine how sulfur availability and environmental conditions shape microbial assemblages across different biofilm types in a cold sulfur spring system at Blount Springs, Alabama (33.9301° N, 86.7928° W). Sulfur‐oxidizing chemolithotrophs, including *Sulfurovum* and *Halothiobacillus*, represented the majority of the recovered reads in sulfur‐rich white biofilms, while purple phototrophic biofilms were enriched with anoxygenic sulfur‐oxidizing bacteria, such as *Chromatium* and *Chlorobium*. Nonsulfur biofilms from adjacent environments displayed greater microbial diversity, including a high abundance of photosynthetic diatoms, like, *Melosira*. Notably, *Sulfurovum* was abundant across both sulfur‐rich and phototrophic niches, suggesting ecological flexibility and a central role in sulfur metabolism. These findings highlight the influence of sulfur chemistry and light availability in structuring microbial communities and contribute to a broader understanding of microbial adaptation and sulfur cycling in cold sulfur spring ecosystems.

## Graphical abstract

Distinct microbial communities occur between adjacent sulfur‐rich and freshwater springs. Chemolithotrophic and phototrophic sulfur‐oxidizing taxa were enriched in cold sulfur spring biofilms, while adjacent freshwater biofilms supported more diverse microbial community composition.

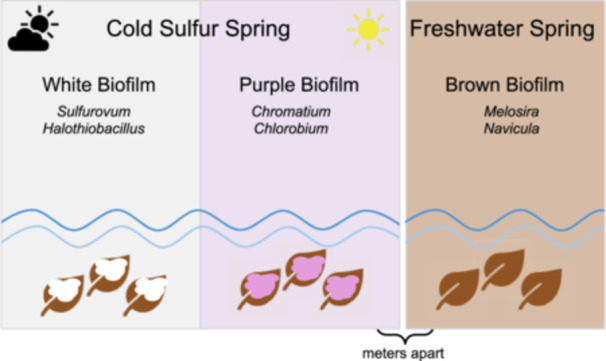

## 1 Introduction

Sulfur‐rich environments support distinctive microbial communities that drive key biogeochemical processes, particularly sulfur cycling (Ward et al. 1998; Rossmassler et al. 2016). These ecosystems occur under a wide range of geochemical and thermal conditions, from hydrothermal vents and volcanic hot springs to sulfidic cave systems and cold terrestrial springs (Skirnisdottir et al. 2000; Macalady et al. 2006; Perreault et al. 2007). Although thermophilic systems have been studied extensively, cold sulfur springs remain comparatively overlooked, despite their potential to harbor diverse and metabolically specialized microbiota (Elshahed et al. 2003; Chaudhary et al. 2009; Magnuson et al. 2021; Nosalova et al. 2023).

The geochemistry of sulfur springs is typically influenced by deep groundwater discharge enriched with reduced sulfur compounds, such as hydrogen sulfide or elemental sulfur (Valentin‐Alvarado et al. 2024). These compounds serve as electron donors for sulfur‐oxidizing bacteria, which catalyze sulfur oxidation and support primary production in low‐organic or light‐limited conditions (Friedrich et al. 2001; Mußmann et al. 2007). Among the dominant lineages in these environments are members of the Proteobacteria and Campylobacterota phyla, both capable of sulfur oxidation under aerobic or microaerobic conditions (Campbell et al. 2006; Hamilton et al. 2014). Within these groups, genera like *Sulfurovum* and *Thiothrix* are frequently encountered and have been identified as important contributors to sulfur cycling in cold, sulfidic habitats (Nosalova et al. 2023).

Community composition in sulfur springs is shaped by gradients in sulfide concentration, oxygen availability, light, and substrate type (Fierer and Jackson 2006; Jones et al. 2012; Swingley et al. 2012). In surface‐exposed zones with access to light, anoxygenic phototrophs such as *Chromatium* and *Chlorobium* can form dense purple mats, contributing to sulfur metabolism while creating layered, stratified microbial habitats (Klatt et al. 2011; Hamilton et al. 2014). Deeper or more shaded areas, where sulfide levels are elevated and oxygen is limited, tend to support white chemolithotrophic biofilms dominated by sulfur‐oxidizing taxa (Macalady et al. 2006; Jones et al. 2012).

Blount Springs, located in northern Alabama, is a cold sulfur spring system with continuous sulfur‐rich flow and visibly distinct microbial mats forming on submerged organic substrates. The springs flow from a sequence of Paleozoic age sedimentary rocks that range from Silurian to Mississippian in age along the eastern limb of the Sequatchie anticline (an upfolded sequence of rocks) (Szabo 1988). The strata in the vicinity of the springs are comprised of various fossiliferous Silurian and Mississippian limestones and a thin unit of Chattanooga Shale. The springs are formed in the Red Mountain Formation, which is the oldest geologic formation in the local strata. The impervious black shale of the Devonian age Chattanooga Shale stratigraphically overlies the limestone, forming a confining layer or aquitard (Faust 1984).

There are two possible sources for the sulfur in the springs. One source is the mineral pyrite (FeS_2_), which is in the Chattanooga Shale. The other possible source is from hydrocarbons that are in the surface outcrops of Mississippian‐age Tuscumbia Limestone. Both of these formations are stratigraphically above the Silurian rocks where the springs emerge. Since the strata are faulted and folded into an anticline, there could be a hydrologic connection to either or both formations.

Sulfur springs in the southeastern United States remain largely uncharacterized, despite widespread distribution. This study offers an opportunity to examine microbial communities associated with three visually and ecologically distinct biofilm types. By comparing chemolithotrophic, phototrophic, and freshwater‐associated biofilms within the same spring system, this study provides new insight regarding the influence of sulfur availability and light on microbial diversity in a nonthermal sulfur spring environment.

## 2 Materials and Methods

### 2.1 Site Description

**Figure 1:**
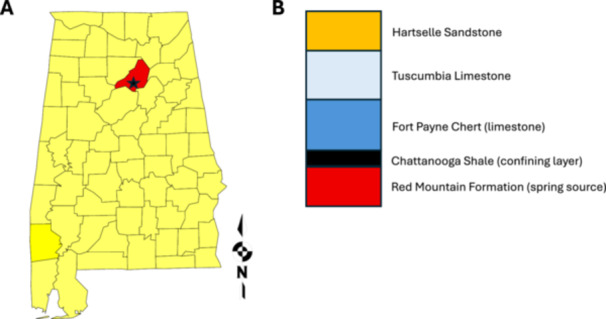
Location and geologic context of Blount Springs, Alabama. (A) County map of Blount County (red) in Alabama, USA Blount Springs location indicated with a star. (B) Generalized stratigraphic relationships of the Paleozoic formations from which Blount Springs emerge. The panel illustrates the major units that influence groundwater flow and potential sources of sulfur.

Samples were collected from Blount Springs, Alabama, a cold (nonthermal) sulfur spring system in northern Alabama (33.9301° N, 86.7928° W) with multiple visible groundwater emergence points along a short stretch of hillside. The seeps emerge from the Red Mountain Formation, which is overlain by Chattanooga Shale acting as a confining layer (Figure 1). The spring discharge then flows across younger Mississippian units, including the Fort Payne Chert, Tuscumbia Limestone, and Hartselle Sandstone. These include several sulfur‐rich spring outlets and at least one adjacent freshwater emergence, all located within approximately a few meters of one another. The outflows converge within a shallow drainage channel lined with submerged leaf litter and microbial mats.

### 2.2 Physicochemical Measurements

Water samples were collected and transported on ice to a certified analytical laboratory for analysis in less than 1 h. Measurements were performed using Standard Methods and Hach‐based assays following manufacturer and method guidelines. Parameters measured included temperature, pH, conductivity, turbidity, total suspended solids, ammonia, nitrate/nitrite, sulfate, alkalinity, and dissolved sulfide.

### 2.3 Biofilm Sampling

Three distinct communities were identified based on coloration on organic substrates. White and purple biofilms were collected from sulfur‐rich areas directly downstream of the spring's source, while brown biofilm samples were obtained from a nearby nonsulfur control spring. Triplicate samples for each biofilm type were swabbed from submerged leaf litter using sterile cotton swabs and immediately submerged in DNA/RNA Shield (Zymo Research), transported on ice, and stored at −20°C until processed.

### 2.4 DNA Extraction and 16S ribosomal RNA (rRNA) Sequencing

Genomic DNA was extracted using the ZymoBIOMICS DNA Miniprep Kit, following the manufacturer's instructions. Amplification of the V3–V4 region of the 16S rRNA gene was performed using the Quick‐16S NGS Library Prep Kit. Amplicons were sequenced on an Illumina NextSeq platform, generating paired‐end reads.

### 2.5 Bioinformatics and Statistical Analysis

Raw sequences were processed using the QIIME pipeline v1.9.1 and DADA2 for denoising and chimera removal. Taxonomic classification was performed using a pretrained classifier against the SILVA database. Heatmaps were generated from log‐transformed relative abundance values to compare genus‐level community structure. Alpha diversity indices (Shannon and Simpson) were calculated to assess within‐sample diversity. Beta diversity was assessed using Bray–Curtis dissimilarity and visualized through Principal Coordinates Analysis (PCoA). Differences in community composition among biofilm types were evaluated using permutational multivariate analysis of variance (PERMANOVA) based on Bray–Curtis distances.

### 2.6 Use of AI Tools

ChatGPT (OpenAI) was used to assist in text editing and to troubleshoot figure design and preparation in R.

## 3 Results

### 3.1 Environmental Parameters and Biofilm Distribution

The main sulfur spring outlet discharged water at 16.4°C with elevated total dissolved solids (TDS = 842 mg/L) and high alkalinity (267 mg/L as CaCO_3_), but no detectable sulfide or sulfate at the source. Although sulfide was below detection limits (BDLs) in the water, microgradients within biofilm or rapid sulfide oxidation can sustain sulfur‐oxidizing taxa even when water measurements read BDL. Downstream from the spring outlet, where sulfur‐associated microbial mats had formed, water temperature remained stable (16.3°C), but sulfate levels increased (20.3 mg/L), suggesting localized sulfur oxidation. The nearby freshwater outlet without sulfur odor or visible mineral deposition (exhibited lower TDS [78 mg/L]), lower alkalinity (80.4 mg/L), and no detectable ammonia, nitrate/nitrite, or sulfate. The pH was slightly alkaline across all sites, ranging from 7.9 at the spring source to 8.23 at the freshwater stream. Ammonia was detectable in the sulfur springs but BDLs in freshwater. Turbidity and total suspended solids (TSS) were elevated at the downstream site, consistent with mat‐associated particulates (Table 1).

**Table 1:** Physicochemical characteristics of spring water at Blount Springs, Alabama.

	Sulfur spring outlet	Downstream	Freshwater emergence
pH	7.9	8.2	8.2
Alkalinity (mg/L)	267	130	180
Nitrate–nitrite (mg/L)	BDL	BDL	BDL
Ammonia (mg/L)	0.33	0.11	BDL
Sulfate, total (mg/L)	BDL	20.3	BDL
Sulfide, total (mg/L)	BDL	BDL	BDL
Turbidity (NTU)	BDL	38	3
TSS (mg/L)	BDL	2	3
TDS (mg/L)	842	336	78

Abbreviations: BDLs, below detection limits; NTU, nephelometric turbidity unit; TDS, total dissolved solids; TSS, total suspended solids.

**Figure 2:**
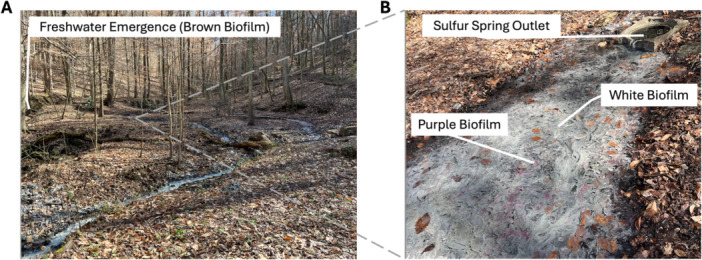
Spatial layout and biofilm types observed at Blount Springs, Alabama. (A) Freshwater emergence, where brown heterogeneous biofilm was collected, and no detectable sulfur odor or deposition. (B) Zoomed‐in view showing sulfur‐rich spring outlet, showing white filamentous sulfur‐oxidizing biofilms and interspersed purple phototrophic mats in sunlit areas.

Three distinct biofilm types were observed within the spring system. White filamentous mats were present from the sulfur‐rich source and extended downstream, coating submerged leaves and woody debris. Purple mats, less cohesive in structure, appeared intermixed with white mats in areas receiving direct sunlight. Both white and purple biofilms were restricted to sulfur‐rich waters. In contrast, brown heterogeneous biofilms formed at the nearby freshwater emergence, where no sulfur odor or visible mineral deposition was detected. Biofilm type appeared linked to sulfur availability and light exposure across microhabitats (Figure 2).

### 3.2 Microbial Community Composition Across Biofilm Types

**Figure 3:**
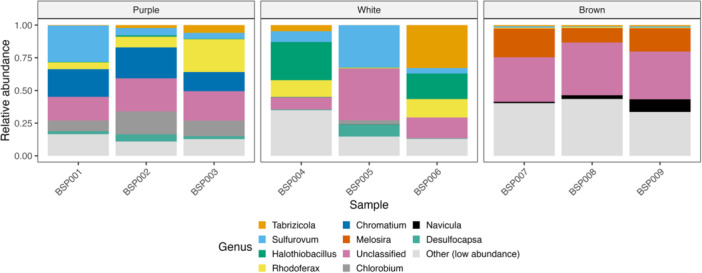
Relative abundance of bacterial genera across biofilm types at Blount Springs. Stacked bar plot showing genus‐level 16S ribosomal RNA sequencing results for individual purple, white, and brown biofilm samples. The 12 most abundant genera are shown; low‐abundance taxa are grouped as “Other.” Colors corresponding to taxonomic groups. The *y*‐axis indicates proportional abundance (0–1).

Genus‐level relative abundances revealed distinct microbial communities across the three biofilm types (Figure 3). In white biofilms, *Sulfurovum* and *Halothiobacillus* comprised a large proportion of recovered reads, consistent with sulfur‐oxidizing metabolism. Purple biofilms included these same sulfur‐oxidizing genera but also included substantial contributions of anoxygenic phototrophs, such as *Chromatium* and *Chlorobium*. In contrast, brown biofilms from the freshwater site exhibited greater taxonomic diversity and included heterotrophic and iron‐reducing genera, such as *Rhodoferax*, *Tabrizicola*, and the diatom *Melosira*, alongside a larger proportion of unclassified bacteria.

**Figure 4:**
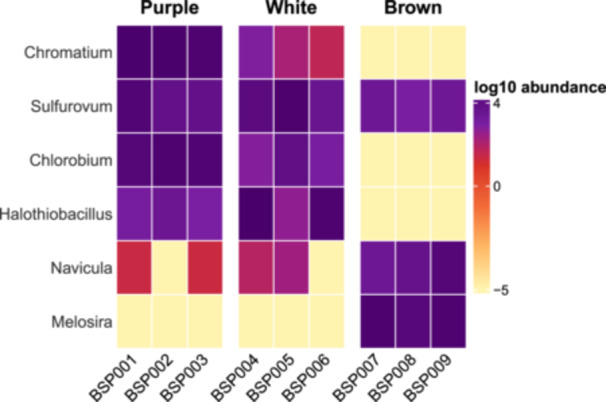
Heatmap of log‐transformed relative abundances of selected bacterial and algal genera across biofilm types. Each column represents an individual sample, and each row corresponds to a genus. Colors indicate log_10_‐transformed relative abundance values. Purple biofilms showed elevated abundances of *Chromatium* and *Chlorobium*, along with sulfur‐associated genera, such as *Sulfurovum* and *Halothiobacillus*, while Brown biofilms were enriched in the diatoms *Navicula* and *Melosira*.

A heatmap of log‐transformed genus‐level abundances revealed clear differences in community composition among the three biofilm types (Figure 4). Purple mats showed elevated abundances of *Chromatium* and *Chlorobium*, together with sulfur‐oxidizing genera, such as *Sulfurovum* and *Halothiobacillus*. White biofilms also contained substantial proportions of *Sulfurovum* and *Halothiobacillus* and fewer phototrophic taxa. In contrast, brown biofilms were characterized by higher abundances of the diatoms *Navicula* and *Melosira* and generally low representation of sulfur‐associated genera. These patterns correspond to gradients in sulfur availability and light exposure across the spring system.

### 3.3 Alpha Diversity Across Biofilm Types

**Figure 5:**
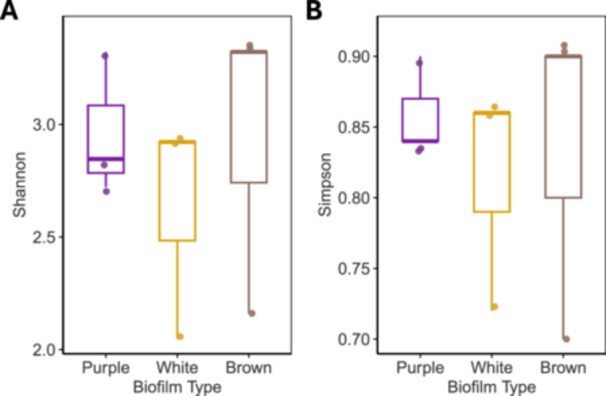
Alpha diversity of microbial communities across biofilm types at Blount Springs. (A) Shannon's diversity values are observed in purple, white, and brown samples. White biofilms showed the lowest Shannon diversity and the widest variability among replicates. (B) Simpson's evenness values for the same biofilm types. Brown and purple biofilms exhibited higher evenness than white mats. Differences among groups were not statistically significant at the *p* < 0.05 level (Shannon *p* = 0.061; Simpson *p* = 0.202).

Alpha diversity varied across the three biofilm types (Figure 5). Shannon diversity was highest in Brown biofilms, with lower values observed in purple and white samples. White biofilms showed the lowest Shannon diversity and widest variability among replicates (Figure 5A). A similar pattern was observed with the Simpson index, where brown and purple biofilms exhibited higher evenness than white mats (Figure 5B). Although the overall trend indicated reduced diversity and evenness in sulfur‐rich samples, differences were not statistically significant at the *p* < 0.05 level (Shannon *p* = 0.061; Simpson *p* = 0.202).

### 3.4 Beta Diversity Reveals Distinct Community Structure by Biofilm Type

**Figure 6:**
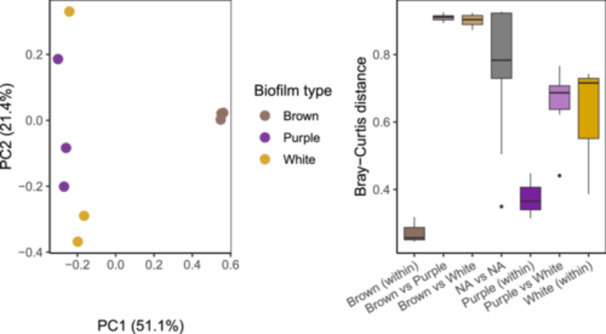
Beta diversity of microbial communities across biofilm types at Blount Springs. (A) Principal Coordinates Analysis of Bray–Curtis dissimilarity showing patterns for each of the three biofilm types. Each point represents an individual sample; axes represent the first two principal coordinates. (B) Boxplots of pairwise Bray–Curtis dissimilarity within and between biofilm types. Between‐group comparisons (e.g., purple vs. brown) show greater dissimilarity than within‐group comparisons. Permutational multivariate analysis of variance indicated significant differences in community composition among biofilm types (*R* ^2^ = 0.728, *p* = 0.002), and multivariate dispersion did not differ significantly among groups (*p* = 0.291).

Beta diversity analysis based on Bray–Curtis dissimilarity showed that microbial communities differed among the three biofilm types (Figure 6). PCoA showed that Brown biofilm samples formed a tight cluster, distinct from white and purple mats, which exhibited some overlap but remained separate from the freshwater group.

Boxplot comparisons of Bray–Curtis distances further illustrated these patterns. Within‐group dissimilarity was lowest in Brown biofilms, intermediate in purple, and highest in white biofilm samples. The highest dissimilarity was observed between sulfur‐associated and nonsulfur biofilms. PERMANOVA confirmed that microbial community composition differed significantly by biofilm type (Bray–Curtis; *R* ^2^ = 0.728, *p* = 0.002). A test of multivariate dispersion indicated no significant difference in within‐group variance (betadisper *p* = 0.291), supporting the interpretation that observed group differences reflect shifts in community structure rather than differences in dispersion.

## 4 Discussion

The microbial community structure in these cold sulfur springs is shaped by fine‐scale environmental gradients, particularly sulfur availability and light exposure. By comparing three distinct biofilm types, white filamentous, purple phototrophic, and brown freshwater‐associated, we found that both taxonomic composition and diversity varied systematically across microhabitats within a single spring system (Figures 1 and 2).

White biofilms were enriched in sulfur‐oxidizing chemolithotrophs, such as *Sulfurovum* and *Halothiobacillus*, taxa commonly associated with sulfide‐rich, microaerobic environments (Campbell et al. 2006; Hamilton et al. 2014). Purple biofilms, found intermixed with white mats in sunlit areas (Figure 2), contained a mix of sulfur‐oxidizers and phototrophic sulfur bacteria, including *Chromatium* and *Chlorobium* (Figure 3), consistent with observations from other stratified systems in sulfidic environments (Klatt et al. 2011; Hamilton et al. 2014). The presence of *Sulfurovum* in both white and purple biofilm (Figure 4) suggests this genus plays a flexible and possibly central role in sulfur cycling in this system, similar to patterns observed in cold sulfidic springs elsewhere (Magnuson et al. 2021; Nosalova et al. 2023).

In contrast, brown biofilms from the adjacent freshwater emergence lacked abundant sulfur‐metabolizing taxa and exhibited a more even assembly of heterotrophs and photosynthetic eukaryotes, like, *Melosira* and *Navicula* (Figure 4). The identification of diatom‐associated sequences and the reduced or absent abundance of sulfur‐oxidizing or phototrophic sulfur bacteria reflects a more oxidized, low‐sulfide condition of a freshwater spring. Alpha diversity metrics (Figure 5) supported this pattern, with brown biofilms showing the highest richness and evenness, while white mats were dominated by a few sulfur‐specialized taxa. This pattern is consistent with environmental filtering in chemically extreme conditions, where redox constraints limit niche availability and taxonomic diversity (Fierer and Jackson 2006; Perreault et al. 2007).

Beta diversity patterns further reinforced this differentiation (Figure 6). White and purple biofilms clustered together based on Bray–Curtis dissimilarity, while brown biofilms formed a distinct group. PERMANOVA confirmed significant differences in community composition among groups, indicating that sulfur availability and freshwater conditions structure these communities. The relatively low dissimilarity within the brown group suggests a more stable community compared with the sulfur‐associated mats, where localized gradients in sulfide and light may create varied microhabitats (Douglas 2001; Rossmassler et al. 2016).

One notable absence from the sulfur‐rich biofilms was *Beggiatoa*, a filamentous sulfur oxidizer commonly reported in cold springs and sulfidic sediments (Macalady et al. 2006; Mußmann et al. 2007). Although dense white filamentous material was observed on submerged surfaces, 16S rRNA sequencing did not detect *Beggiatoa* or other known filamentous sulfur‐oxidizing genera, such as *Thiothrix*. The filaments may instead represent dense biofilms dominated by nonfilamentous sulfur oxidizers, such as *Sulfurovum* and *Halothiobacillus*, or possibly abiotic sulfur accumulation associated with microbial activity (Sharrar et al. 2017). Without direct verification, the identity of the filaments remains uncertain, but their presence highlights the potential for diverse physical biofilm structures in sulfur‐rich environments, even in the absence of classical filamentous taxa.

## 5 Conclusions

Together, this study highlights the complexity of cold sulfur spring environments and the strong spatial structuring of microbial communities over short distances. Although cold sulfidic springs are less studied than their geothermal counterparts, they may host similarly specialized and diverse microbiota (Perreault et al. 2007; Nosalova et al. 2023). The combination of chemolithotrophic and phototrophic sulfur metabolism observed here underscores the importance of light‐sulfide interactions in shaping microbial community assembly (Valentin‐Alvarado et al. 2024).

Future work should include targeted metagenomic or metatranscriptomic analyses to resolve functional pathways of sulfur metabolism in situ. Additionally, high‐resolution measurements of sulfide and oxygen gradients within the mats, along with seasonal sampling, could clarify the temporal and spatial dynamics of community structure (Inskeep et al. 2010; Swingley et al. 2012). Given the proximity of freshwater and sulfur‐rich microhabitats at Blount Springs, this site offers a unique natural laboratory for exploring microbial adaptation, niche partitioning, and the evolution of sulfur‐based energy metabolisms in low‐temperature environments.

## Author Contributions

**Kevin M. Drace:** conceptualization, methodology, investigation, formal analysis, validation, data curation, visualization, writing, review and editing, supervision, project administration, resources. **David M. Frings:** writing, review and editing, visualization. **James M. Mellinger:** investigation.

## Funding

The authors received no specific funding for this work.

## Ethics Statement

The authors have nothing to report.

## Conflicts of Interest

The authors declare no conflicts of interest.

## Data Availability

Raw sequence data generated from this study have been deposited in the NCBI Sequence Read Archive (SRA) under BioProject accession PRJNA1291808.

## References

[mbo370223-bib-0001] Campbell, B. J. , A. S. Engel , M. L. Porter , and K. Takai . 2006. “The Versatile ε‐Proteobacteria: Key Players in Sulphidic Habitats.” Nature Reviews Microbiology 4, no. 6: 458–468. 10.1038/nrmicro1414. 16652138

[mbo370223-bib-0002] Chaudhary, A. , S. K. Haack , J. W. Duris , and T. L. Marsh . 2009. “Bacterial and Archaeal Phylogenetic Diversity of a Cold Sulfur‐Rich Spring on the Shoreline of Lake Erie, Michigan.” Applied and Environmental Microbiology 75, no. 15: 5025–5036. 10.1128/AEM.00112-09. 19542341 PMC2725501

[mbo370223-bib-0003] Douglas, S. D. D. 2001. “Structural and Geomicrobiological Characteristics of a Microbial Community From a Cold Sulfide Spring.” Geomicrobiology Journal 18, no. 4: 401–422. 10.1080/014904501753210567.

[mbo370223-bib-0004] Elshahed, M. S. , J. M. Senko , F. Z. Najar , et al. 2003. “Bacterial Diversity and Sulfur Cycling in a Mesophilic Sulfide‐Rich Spring.” Applied and Environmental Microbiology 69, no. 9: 5609–5621. 10.1128/AEM.69.9.5609-5621.2003. 12957951 PMC194924

[mbo370223-bib-0005] Faust, R. J. 1984. Geology of Blount County, Alabama. Geological Survey of Alabama (Special Map 159).

[mbo370223-bib-0006] Fierer, N. , and R. B. Jackson . 2006. “The Diversity and Biogeography of Soil Bacterial Communities.” Proceedings of the National Academy of Sciences 103, no. 3: 626–631. 10.1073/pnas.0507535103. PMC1334650 16407148

[mbo370223-bib-0007] Friedrich, C. G. , D. Rother , F. Bardischewsky , A. Quentmeier , and J. Fischer . 2001. “Oxidation of Reduced Inorganic Sulfur Compounds by Bacteria: Emergence of a Common Mechanism?” Applied and Environmental Microbiology 67, no. 7: 2873–2882. 10.1128/AEM.67.7.2873-2882.2001. 11425697 PMC92956

[mbo370223-bib-0008] Hamilton, T. L. , R. J. Bovee , V. Thiel , et al. 2014. “Coupled Reductive and Oxidative Sulfur Cycling in the Phototrophic Plate of a Meromictic Lake.” Geobiology 12, no. 5: 451–468. 10.1111/gbi.12092. 24976102

[mbo370223-bib-0009] Inskeep, W. P. , D. B. Rusch , Z. J. Jay , et al. 2010. “Metagenomes From High‐Temperature Chemotrophic Systems Reveal Geochemical Controls on Microbial Community Structure and Function.” PLoS ONE 5, no. 3: e9773. 10.1371/journal.pone.0009773. 20333304 PMC2841643

[mbo370223-bib-0010] Jones, D. S. , H. L. Albrecht , K. S. Dawson , et al. 2012. “Community Genomic Analysis of an Extremely Acidophilic Sulfur‐Oxidizing Biofilm.” ISME Journal 6, no. 1: 158–170. 10.1038/ismej.2011.75. 21716305 PMC3246232

[mbo370223-bib-0011] Klatt, C. G. , J. M. Wood , D. B. Rusch , et al. 2011. “Community Ecology of Hot Spring Cyanobacterial Mats: Predominant Populations and Their Functional Potential.” ISME Journal 5, no. 8: 1262–1278. 10.1038/ismej.2011.73. 21697961 PMC3146275

[mbo370223-bib-0012] Macalady, J. L. , E. H. Lyon , B. Koffman , et al. 2006. “Dominant Microbial Populations in Limestone‐Corroding Stream Biofilms, Frasassi Cave System, Italy.” Applied and Environmental Microbiology 72, no. 8: 5596–5609. 10.1128/AEM.00715-06. 16885314 PMC1538711

[mbo370223-bib-0013] Magnuson, E. , N. C. S. Mykytczuk , A. Pellerin , et al. 2021. “ *Thiomicrorhabdus* Streamers and Sulfur Cycling in Perennial Hypersaline Cold Springs in the Canadian High Arctic.” Environmental Microbiology 23, no. 7: 3384–3400. 10.1111/1462-2920.14916. 31943734

[mbo370223-bib-0014] Mußmann, M. , F. Z. Hu , M. Richter , et al. 2007. “Insights into the Genome of Large Sulfur Bacteria Revealed by Analysis of Single Filaments.” PLoS Biology 5, no. 9: e230. 10.1371/journal.pbio.0050230. 17760503 PMC1951784

[mbo370223-bib-0015] Nosalova, L. , C. Mekadim , J. Mrazek , and P. Pristas . 2023. “ *Thiothrix* and *Sulfurovum* Genera Dominate Bacterial Mats in Slovak Cold Sulfur Springs.” Environmental Microbiome 18, no. 1: 72. 10.1186/s40793-023-00527-4. 37730677 PMC10512639

[mbo370223-bib-0016] Perreault, N. N. , D. T. Andersen , W. H. Pollard , C. W. Greer , and L. G. Whyte . 2007. “Characterization of the Prokaryotic Diversity in Cold Saline Perennial Springs of the Canadian High Arctic.” Applied and Environmental Microbiology 73, no. 5: 1532–1543. 10.1128/AEM.01729-06. 17220254 PMC1828757

[mbo370223-bib-0017] Rossmassler, K. , T. E. Hanson , and B. J. Campbell . 2016. “Diverse Sulfur Metabolisms From Two Subterranean Sulfidic Spring Systems.” FEMS Microbiology Letters 363, no. 16: fnw162. 10.1093/femsle/fnw162. 27324397

[mbo370223-bib-0018] Sharrar, A. M. , B. E. Flood , J. V. Bailey , et al. 2017. “Novel Large Sulfur Bacteria in the Metagenomes of Groundwater‐Fed Chemosynthetic Microbial Mats in the Lake Huron Basin.” Frontiers in Microbiology 8: 791. 10.3389/fmicb.2017.00791. 28533768 PMC5421297

[mbo370223-bib-0019] Skirnisdottir, S. , G. O. Hreggvidsson , S. Hjörleifsdottir , et al. 2000. “Influence of Sulfide and Temperature on Species Composition and Community Structure of Hot Spring Microbial Mats.” Applied and Environmental Microbiology 66, no. 7: 2835–2841. 10.1128/AEM.66.7.2835-2841.2000. 10877776 PMC92081

[mbo370223-bib-0020] Swingley, W. D. , D. R. Meyer‐Dombard , E. L. Shock , et al. 2012. “Coordinating Environmental Genomics and Geochemistry Reveals Metabolic Transitions in a Hot Spring Ecosystem.” PLoS ONE 7, no. 6: e38108. 10.1371/journal.pone.0038108. 22675512 PMC3367023

[mbo370223-bib-0021] Szabo, M. W. , E. W. Osborne , C. W. Copeland , and T. L. Neathery . 1988. Geologic Map of Alabama. Geological Survey of Alabama.

[mbo370223-bib-0022] Valentin‐Alvarado, L. E. , S. C. Fakra , A. J. Probst , et al. 2024. “Autotrophic Biofilms Sustained by Deeply Sourced Groundwater Host Diverse Bacteria Implicated in Sulfur and Hydrogen Metabolism.” Microbiome 12, no. 1: 15. 10.1186/s40168-023-01704-w. 38273328 PMC10811913

[mbo370223-bib-0023] Ward, D. M. , M. J. Ferris , S. C. Nold , and M. M. Bateson . 1998. “A Natural View of Microbial Biodiversity Within Hot Spring Cyanobacterial Mat Communities.” Microbiology and Molecular Biology Reviews 62, no. 4: 1353–1370. 10.1128/MMBR.62.4.1353-1370.1998. 9841675 PMC98949

